# Acute kidney injury as a predictor of infectious complications after mini‐PCNL

**DOI:** 10.1002/bco2.70084

**Published:** 2025-09-07

**Authors:** Angelo Cormio, Daniele Castellani, Domenico De Palma, Ruggiero Fiorella, Runeel Ratnayake, Michele Lotito, Giuseppe Albino, Ugo Giovanni Falagario, Gian Maria Busetto, Carlo Bettocchi, Giuseppe Carrieri, Luigi Cormio

**Affiliations:** ^1^ Urology Unit Azienda Ospedaliero‐Universitaria delle Marche Ancona Italy; ^2^ Department of Urology and Renal Transplantation, Policlinico Riuniti di Foggia University of Foggia Foggia Italy; ^3^ Endourology Section of the European Association of Urology Arnhem The Netherlands; ^4^ Department of Urology and Andrology, Bonomo Teaching Hospital Andria Italy

**Keywords:** acute kidney injury, infectious complications, kidney calculi, percutaneous nephrolithotomy

## Abstract

**Objective:**

To investigate the incidence, risk factors and clinical consequences of acute kidney injury (AKI) following mini‐percutaneous nephrolithotomy (mini‐PCNL), with particular focus on its association with postoperative infectious complications.

**Materials and Methods:**

A retrospective analysis was conducted on 496 adult patients who underwent mini‐PCNL (22 Ch) between February 2020 and April 2025. AKI was defined according to KDIGO criteria as either a ≥ 1.5‐fold increase or an absolute increase of ≥0.3 mg/dl in serum creatinine within 72 hours postoperatively. Patients were stratified into AKI and non‐AKI groups. Multivariable logistic regression analyses were performed to identify predictors of AKI development and infectious complications.

**Results:**

Surgery was done in spinal anaesthesia in all cases. AKI occurred in 45 patients (9.1%). There was no difference in median surgical time (52.5 vs 55.0 minutes, p = 0.33) between groups. There was no difference between the two groups in gender distribution, median age, body mass index, baseline serum creatinine, rates of comorbidities and stone features. Patients with AKI had significantly higher rates of overall postoperative complications (24.4% vs 7.1%, p < 0.001) and longer hospital stays (4 vs 3 days, p < 0.001). Infectious complications were significantly more frequent in the AKI group, with higher median procalcitonin levels (0.21 vs 0.06 ng/ml, p = 0.03). One patient in the AKI group died from sepsis. Multivariable analysis identified previous PCNL (OR 2.51, 95% CI 1.33–4.72, p < 0.01) and higher baseline serum creatinine (OR 2.00, 95% CI 1.07–3.73, p = 0.03) as independent predictors of AKI. AKI was the only independent predictor of infectious complications (OR 3.47, 95% CI 1.04–11.58, p = 0.04).

**Conclusions:**

The strong association between AKI and infectious complications, including potential mortality from sepsis, highlights the clinical significance of this underreported complication. Enhanced perioperative monitoring and aggressive management of infectious complications are warranted in patients who develop AKI following mini‐PCNL.

## 1 INTRODUCTION

According to the European Association of Urology and American Urological Association/Endourological Society guidelines, percutaneous nephrolithotripsy (PCNL) remains the first option for complex kidney stones or stones larger than 2 cm.^1^, ^2^ Traditionally, the size of the Amplatz sheath in standard (s)‐PCNL was 30 Ch; however, decreasing the size of the percutaneous tract has been shown to reduce complication rates without compromising stone‐free outcomes when compared to s‐PCNL techniques.^3^ As a result, miniaturized (mini)‐PCNL, generally characterized by a tract diameter of less than 22 Ch, has become widely adopted and is now commonly used for managing stones larger than 2 cm^4^ and in obese patients.^5^

Acute kidney injury (AKI) is an underreported yet significant postoperative complication associated with endourological procedures for renal stones that can negatively impact patient outcomes. According to the Kidney Disease: Improving Global Outcomes (KDIGO) criteria, AKI is diagnosed based on changes in serum creatinine levels, defined as an increase of ≥0.3 mg/dl or more than 1.5 times the baseline value, measured from the preoperative (baseline) period to the early postoperative period (24 to 72 hours following the procedure).^6^ Furthermore, individuals who develop AKI are at heightened risk of progressing to CKD, a condition linked to substantial economic, social and personal burdens, as well as increased mortality risk.^7^ This association is particularly significant in patients with a solitary kidney and/or preexisting chronic kidney disease (CKD).

The incidence of AKI following PCNL has been reported to be 13.9%^8^ with rates reaching up to 25% in patients with a solitary kidney.^9^ The aetiology of post‐PCNL AKI is likely multifactorial. Loss of nephrons due to the trauma caused by puncturing and dilating the renal parenchyma is commonly viewed as a relevant risk factor, but several preoperative factors, such as comorbidities and baseline renal function, as well as intraoperative conditions, such as high intrapelvic pressures and release of bacteria during stone fragmentation, may all play relevant roles. A comprehensive understanding of AKI predictors and clinical sequelae is therefore eagerly awaited to optimize patient care and surgical outcomes. This understanding is crucial, as up to 17.2% of patients with post‐PCNL AKI may develop chronic kidney disease.^8^

To the best of our knowledge, only one study has reported the incidence of AKI following mini‐PCNL.^10^ The present study aimed to characterize the incidence, risk factors and clinical consequences of AKI following mini‐PCNL for renal stones, with particular attention to its correlation with postoperative infectious complications.

## 2 METHODS

A retrospective analysis was conducted using data from our institutional review board‐approved PCNL database, specifically including adult patients who underwent mini‐PCNL between February 2020 and April 2025. Inclusion was limited to adults with renal calculi. The exclusion criteria comprised paediatric/adolescent patients, concomitant ureteral stones, malignant renal tumours, bilateral procedures and stones located in a calyceal diverticulum.

All patients underwent a preoperative abdominal computed tomography (CT) scan to assess stone burden, including number, size and location. Stone size was recorded as the longest diameter for single stones or the cumulative diameter in cases of multiple calculi. Antiplatelet and anticoagulant therapy was discontinued 3–7 days before surgery, depending on individual clinical circumstances. Renal function was evaluated using serum creatinine levels obtained preoperatively (baseline) and at 24 and 72 hours postoperatively. AKI was defined according to the KDIGO criteria as either a ≥ 1.5‐fold increase or an absolute increase of ≥0.3 mg/dl in serum creatinine within 72 hours postoperatively.^6^

Midstream urine cultures were obtained preoperatively to screen for urinary tract infections. Patients with positive cultures received pathogen‐specific antibiotic treatment to achieve urinary sterilization; if cultures remained positive, targeted antibiotic therapy was initiated three days before the procedure. Patients with negative cultures received prophylactic intravenous antibiotics with 2 g of cefazolin and 300 mg of netilmicin administered 30 minutes before anaesthesia induction, followed by daily intravenous ceftriaxone (1 g) until discharge, provided no infectious complications occurred.

All procedures were performed under spinal anaesthesia, either by or under the supervision of an experienced endourologist (L.C.). Surgical positioning included either the supine anterolateral or the Galdakao‐modified supine Valdivia position.^11^, ^12^ Retrograde pyelography was used to opacify the collecting system, and access was achieved using an 18‐G needle under fluoroscopic guidance, with adjunctive use of ultrasound or endovision technique when appropriate. The calyx targeted for access was selected based on stone characteristics and renal anatomy.

Mini‐PCNL was performed using the Karl Storz MIP system (Tuttlingen, Germany). A single‐step dilation with an 11 Fr metallic dilator was followed by the placement of a 17.5 Fr Amplatz sheath. A 4‐mm rigid nephroscope (Karl Storz) was used for stone visualization and retrieval. Lithotripsy was achieved using either a Holmium: YAG laser (Lumenis, Yokneam, Israel) or a Thulium fibre laser (Quanta System, Varese, Italy), with laser settings optimized for dusting and producing 1–3 mm fragments extractable via the Venturi effect. Completion of stone clearance was confirmed in all cases by flexible nephroscopy or ureteroscopy. Intraoperative stone‐free status, defined as the complete absence of residual fragments, was assessed visually and fluoroscopically.

A tubeless approach with either single‐ or double‐J ureteral stent placement was adopted as the standard exit strategy. Nephrostomy tubes were placed only in cases with significant haemorrhage or elevated infection risk. Stone cultures (SC) were obtained by fragmenting the largest retrieved stones in sterile saline and streaking them onto agar plates.

Postoperative complications occurring within 30 days were categorized according to the Clavien‐Dindo classification adapted for PCNL.^13^ Ethics committee approval was not required, as the data were collected for clinical purposes and all procedures were part of routine care. The study adhered to the ethical principles of the Declaration of Helsinki and its subsequent amendments.

## 3 STATISTICAL ANALYSIS

Patients were stratified into two groups based on the occurrence of postoperative AKI: Group 1 (patients having AKI) and Group 2 (patients without AKI). Continuous variables are reported as medians with interquartile ranges (IQRs), while categorical variables are expressed as absolute counts and percentages. The chi‐square test was used for categorical comparisons between groups, and the Mann–Whitney U test for continuous variables. Two multivariable backwards logistic regression analyses were performed, one to identify predictors of developing postoperative AKI and the other to identify predictors of developing infectious complications, with results reported as odds ratios (OR) with 95% confidence intervals (CI) and corresponding p‐values. Statistical significance was set at a two‐tailed p‐value <0.05. Analyses were performed using SPSS version 25.0 (IBM Corp., Armonk, NY).

## 4 RESULTS

A total of 496 adult patients were included in the analysis; among them, 45 (9.1%) developed postoperative AKI, while 451 (90.9%) did not (Table 1).

**TABLE 1:** Patient baseline characteristics.

	Group 1 (AKI) (n = 45)	Group 2 (no‐AKI) (n = 451)	P value
**Age**, years, median (IQR)	61.8 (34.9)	55.6 (22.1)	0.70
**Gender**			0.45
Male, n (%)	25 (44.4)	227 (50.3)	
Female, n (%)	20 (55.6)	224 (49.7)	
**BMI**, median (IQR)	28.0 (4.4)	26.8 (7.0)	0.38
**Hypertension**, n (%)	15 (33.3)	192 (42.6)	0.23
**Diabetes**, n (%)	7 (15.6)	67 (14.9)	0.90
**ASA score**, n (%)			0.44
1	4 (8.9)	42 (9.3)	
2	28 (62.3)	260 (57.6)	
3	11 (24.4)	142 (31.5)	
4	2 (4.4)	7 (1.6)	
**Preoperative urine culture**, n (%)			0.14
Sterile	36 (80.0)	396 (87.8)	
Positive	9 (20.0)	55 (12.2)	
**Stone laterality**, n (%)			0.88
Right	21 (46.7)	213 (47.2)	
Left	24 (53.3)	238 (52.8)	
**Guys' score**, n (%)			0.12
1	21 (46.7)	216 (47.9)	
2	14 (31.1)	84 (18.6)	
3	6 (13.3)	116 (25.7)	
4	4 (8.9)	35 (7.8)	
**Maximal stone length**, mm, median (IQR)	26.0 (40.0)	22.0 (15.0)	0.36
**Pre‐operative creatinine**, mg/dl, median (IQR)	0.74 (0.15)	0.81 (0.20)	0.53
**Multiple stones**, n (%)	24 (53.3)	212 (47.0)	0.71
**Preoperative stent**, n (%)	13 (28.9)	68 (15.1)	**0.04**
**Preoperative stent dwelling time, months,** median (IQR)	5.0 (5.0)	5.0 (5.0)	0.66
**Previous PCNL**, n (%)	240 (53.3)	146 (32.4)	**0.04**
**Anomalous kidney**, n (%)	3 (6.7)	8 (2.7)	**0.001**
Duplex system	0	5 (1.1)	
Horseshoe kidney	3 (6.7)	2 (0.4)	
Malrotated kidney	0	1 (0.2)	

Abbreviations: **AKI**: Acute Kidney Injury. **ASA**: American Society of Anesthesiologists. **BMI**: Body Mass Index. **IQR**: interquartile range. **PCNL**: percutaneous nephrolithotripsy.

There was no difference between the two groups in gender distribution, median age, body mass index, baseline serum creatinine, as well as in the rates of comorbidities such as hypertension and diabetes mellitus. Stone features did not impact on AKI occurrence, as there was no difference in maximal stone length (26.0 mm vs 22.0 mm, p = 0.36), presence of multiple stones (53.3% vs 47.0%, p = 0.71) and Guy's stone score distribution (p = 0.12).

Conversely, preoperative factors showing significant associations with AKI development included having a preoperative ureteral stent (28.9% vs 15.1%, p = 0.04), previous PCNL (53.3% vs 32.4%, p = 0.04), and having anatomical kidney anomalies (6.7% vs 2.7%, p = 0.001), with horseshoe kidney being the most common anomaly in AKI patients (6.7% vs 0.4%).

Table 2 shows intra‐ and postoperative outcomes. There was no difference in median surgical time (52.5 vs 55.0 minutes, p = 0.33), intraoperative SFR (88.9% vs. 94.7%, p = 0.11) and exit strategy distribution (p = 0.81) among the two groups. As expected, AKI patients experienced significantly higher postoperative serum creatinine levels (1.33 vs 0.82 mg/dl, p < 0.001) and significantly longer hospital stay (4 vs 3 days, p < 0.001).

**TABLE 2:** Intraoperative and postoperative outcomes.

	Group 1 (AKI) (n = 45)	Group 2 (no‐AKI) (n = 451)	P value
**Total surgical time**, minutes, median (IQR)	52.5 (27.9)	55.0 (30)	0.33
**Postoperative creatinine**, mg/dl, median (IQR)	1.33 (1.0)	0.82 (0.3)	**0.001**
**Postoperative stay**, days, median (IQR)	4 (3.0)	3.0 (2.0)	**0.001**
**Exit strategy**, n (%)			0.81
Totally tubeless	0	0	
Double J stent	13 (28.9)	127 (28.2)	
Double J stent and nephrostomy tube	2 (4.4)	27 (6.0)	
Mono J ureteral catheter	27 (60.0)	278 (61.6)	
Mono J ureteral catheter and nephrostomy tube	3 (6.7)	16 (3.5)	
Nephrostomy tube	0	3 (0.7)	
**Intraoperative stone‐free**, n (%)	40 (88.9)	427 (94.7)	0.11
**Positive stone culture**, n (%)	11 (24.4)	70 (15.5)	0.12
**Postoperative complications** ^§^, n (%)			**<0.001**
None	34 (75.6)	419 (92.9)	
***Clavien grade 1***	**5 (11.1)**	**21 (4.7)**	
Postoperative fever (>38.0 C) managed by observation without antibiotics	1 (2.2)	9 (2.0)	
Bleeding managed using intravenous fluid without need for blood transfusion	4 (8.9)	12 (26.7)	
***Clavien grade 2***	**5 (11.1)**	**10 (2.0)**	
Hematuria requiring blood transfusion	1 (2.2)	2 (0.4)	
Postoperative fever (>38.0 C) managed with antibiotics in the ward	4 (8.9)	8 (1.8)	
***Clavien 3a***			
Febrile UTI without organ failure requiring supportive therapy and enhanced monitoring	0	1 (0.2)	
***Clavien 5***			
Death due to sepsis	1 (2.3)	0	
**Infectious complications**			**0.001**
***Clavien grade 1***			
Postoperative fever (>38.0 C) managed by observation without antibiotics	1 (2.2)	9 (2.0)	
***Clavien grade 2***			
Postoperative fever (>38.0 C) managed with antibiotics in the ward)	4 (8.9)	8 (1.8)	
***Clavien 3a***			
Febrile UTI without organ failure requiring supportive therapy and enhanced monitoring	0	1 (0.2)	
***Clavien 5***			
Death due to sepsis	1 (2.3)	0	
**Procalcitonin**, ng/mL, median (IQR)	0.21 (3.30)	0.06 (0.41)	**0.03**

Abbreviations: **AKI**: Acute Kidney Injury. **IQR**: interquartile range. **UTI:** urinary tract infection.

^§^ More than one complication for single patients included.

Overall postoperative complications were significantly more frequent in the AKI group (24.4% vs 7.1%, p < 0.001), particularly Clavien grade 2 (11.1% vs 2.0%) and Clavien grade 3a complications (8.9% vs 1.8%). One patient in the AKI group died due to sepsis (Clavien grade 5), while no deaths occurred in the non‐AKI group.

Infectious complications were significantly more common in patients with AKI (p < 0.001). There were higher rates of positive SC in the AKI group, but this difference was not statistically significant (24.4% vs 15.5%, p = 0.12), whereas median procalcitonin levels were significantly higher in the AKI group (0.21 vs 0.06 ng/ml, p = 0.03).

Multivariable logistic regression analysis testing potential predictors of postoperative AKI (Table 3) showed that previous PCNL was associated with a 2.51‐fold increased risk (OR 2.51, 95% CI 1.33–4.72, p < 0.01) and baseline serum creatinine level was associated with a 2.00‐fold increased risk per unit (OR 2.00, 95% CI 1.07–3.73, p = 0.03). On the other hand, multivariable logistic analysis testing potential predictors of infectious complications showed that AKI was the only independent factor associated with higher odds (OR 3.47 95% CI 1.04–11.58, p = 0.04) of having such complications (Table 4).

**TABLE 3:** Multivariable analysis of predictive factors of having postoperative acute kidney injury.

	OR (95% CI)	*p‐value*
Female gender (reference: male gender)	1.42 (0.75–2.69)	0.28
Previous percutaneous nephrolithotripsy (reference: NO)	2.51 (1.33–4.72)	**<0.01**
Hypertension (reference: NO)	0.66 (0.33–1.31)	0.27
Preoperative serum creatinine	2.00 (1.07–3.73)	**0.03**

**TABLE 4:** Multivariable analysis of predictive factors of having infectious complications.

	OR (95% CI)	*p‐value*
Surgical time	1.01 (0.99–1.03)	0.11
Urine culture (reference: negative)	0.31 (0.04–2,56)	0.28
Stone culture (reference: negative)	0.25 (0.03–2.00)	0.19
AKI	3.47 (1.04–11.58)	**0.04**

## 5 DISCUSSION

The present study revealed that the incidence of AKI after mini‐PCNL was 9.1% and provided insights into its mechanisms and clinical implications, thus contributing to the growing body of literature exploring this complication of endourological procedures.

The incidence of AKI observed in our study is notably higher than the 4.4% reported by Fulla et al. in their analysis of s‐PCNL procedures.^14^ However, our findings align more closely with the broader literature on post‐PCNL AKI, where incidence rates have been reported to range from 10% to 25%, with higher rates typically observed in more complex cases or patients with solitary kidneys.^9^, ^15^ The discrepancy between our results and some published studies may be attributed to several factors, including patient selection criteria and the specific characteristics of our patient population. Several factors may indeed play a role in the development of such complication. The tract size is generally considered one of them, but a recent randomized study on 75 patients^10^ comparing the impact of s‐PCNL (30 Fr), mini‐PCNL (16 Fr) and retrograde intrarenal surgery (with a 11/13 Fr or 12/14 Fr ureteral sheath) on early urinary biomarkers of renal injury (neutrophil gelatinase‐associated lipocalin, kidney injury molecule‐1 and interleukin‐18, all normalized to urinary creatinine) showed that all three procedures caused a significant but similar early increase in urinary biomarkers of tubular injury 2 hours after surgery, indicating some degree of acute renal damage. However, there were no significant differences between the groups in the magnitude of these biomarker changes at any time point and in the incidence of AKI.

That said, it is evident that post‐PCNL AKI has a multifactorial aetiology. Our multivariable analysis identified two independent predictors of postoperative AKI: previous PCNL and higher preoperative serum creatinine levels. These findings are consistent with established risk factors identified in the literature. The association between previous stone treatment and AKI development likely reflects the cumulative nephrotoxic effects of repeated interventions on renal parenchyma. Scarring and fibrosis from previous procedures may compromise nephron function and reduce the kidney's ability to tolerate additional surgical trauma. This finding emphasizes the importance of optimizing stone‐free rates in initial procedures to minimize the need for repeat interventions. The predictive value of preoperative serum creatinine aligns with findings from multiple studies. Fulla et al. identified baseline creatinine >1.54 mg/dl as a significant risk factor for AKI (OR 2.66),^14^ while other studies have consistently demonstrated that pre‐existing renal impairment increases the risk of further kidney injury not only after PCNL^16^ but also following retrograde intrarenal surgery.^17^ The relationship between baseline renal function and post‐procedural AKI underscores the importance of careful perioperative management in patients with compromised kidney function because the clinical significance of post‐procedural AKI extends beyond the immediate perioperative period, as previous studies have demonstrated that up to 17.2% of patients with post‐PCNL AKI may progress to chronic kidney disease.^8^ Interestingly, our study did not identify several traditional risk factors commonly associated with AKI in s‐PCNL, including age, diabetes mellitus, hypertension or stone complexity. This finding may suggest that the reduced tract size in mini‐PCNL could potentially mitigate some of the traditional risk factors associated with renal injury, though this hypothesis requires further investigation in comparative studies.

One of the most significant findings of our study is the strong association between AKI and postoperative infectious complications. Patients who developed AKI had a 3.47‐fold increased risk of infectious complications, representing the only independent predictor of infection in our multivariable analysis. This finding has important clinical implications and aligns with the pathophysiological understanding of AKI's impact on immune function. The relationship between AKI and increased infection risk is multifactorial. AKI can impair immune function through toxin accumulation, alter white blood cell function and create a pro‐inflammatory state that predisposes patients to infectious complications.^18^ The significantly elevated procalcitonin levels in the AKI group support the presence of a heightened inflammatory response in these patients. Additionally, AKI may lead to fluid retention and electrolyte imbalances that can compromise host defence mechanisms.^19^

The significantly higher rate of infectious complications in AKI patients (24.4% vs 7.1%) observed in our study is concerning, particularly given that one patient in the AKI group died from sepsis. This mortality case highlights the potential severity of infectious complications in patients who develop post‐procedural AKI and emphasizes the need for enhanced monitoring. As a consequence, the identification of AKI risk factors in mini‐PCNL has several relevant clinical implications. First, patients with a history of previous PCNL or elevated baseline creatinine should be counselled about their increased risk of AKI and monitored more closely during the perioperative period. Enhanced fluid management, avoidance of nephrotoxic medications, and consideration of alternative treatment approaches may be warranted in high‐risk patients.

Second, the strong association between AKI and infectious complications suggests that patients who develop post‐procedural AKI require heightened surveillance for signs of infection and reinforces the need to collect not only a preoperative mid‐stream urine culture but also an intraoperative SC because the latter better predicts the possibility of developing postoperative sepsis.^20^ Third, our findings support the importance of optimizing stone‐free rates in initial procedures to minimize the need for repeat interventions.

Several limitations of this study should be acknowledged. The retrospective design limits our ability to establish definitive causal relationships and may introduce selection bias. The single‐centre nature of the study may limit generalizability to other institutions with different patient populations or surgical techniques. Additionally, long‐term follow‐up data on the progression of AKI to chronic kidney disease were not available in this analysis. Future research should focus on prospective comparative studies between mini‐PCNL and s‐PCNL to better define the relative risks and benefits of each approach. Investigation of protective strategies for high‐risk patients, such as optimized fluid management protocols or nephroprotective agents, may help reduce the incidence of AKI. Additionally, studies examining the long‐term renal function of patients who develop AKI following mini‐PCNL are needed to fully understand the clinical significance of this complication.

## 6 CONCLUSION

This study shows that AKI occurs in 9.1% of patients undergoing mini‐PCNL, with previous PCNL and elevated baseline serum creatinine as independent risk factors. The strong association between AKI and infectious complications, including one death from sepsis, highlights the potential clinical significance of this neglected complication. These findings emphasize the importance of careful patient selection, enhanced perioperative monitoring and aggressive management of infectious complications in patients who develop AKI following mini‐PCNL. As mini‐PCNL continues to gain acceptance as a preferred approach for managing larger renal stones, understanding and mitigating the risk of AKI will be crucial for optimizing patient outcomes and safety.

## AUTHOR CONTRIBUTIONS

*Study conception*: Luigi Cormio and Daniele Catellani. *Data collection*: Domenico De Palma, Ruggiero Fiorella, Runeel Ratnayake, Michele Lotito, Giuseppe Albino, Ugo Giovanni Falagario, Gian Maria Busetto, and Carlo Bettocchi. *Statistical analysis*: Daniele Castellani. *Manuscript writing*: Angelo Cormio, Daniele Castellani, and Luigi Cormio. *Manuscript review*: Giuseppe Carrieri. *Clinical supervision*: Luigi Cormio and Giuseppe Carrieri.

## CONFLICT OF INTEREST STATEMENT

Daniele Castellani certifies that all conflicts of interest, including specific financial interests and relationships and affiliations relevant to the subject matter or materials discussed in the manuscript (e.g., employment/affiliation, grants or funding, consultancies, honoraria, stock ownership or options, expert testimony, royalties, patents filed, received or pending), are the following: The authors declare no conflict of interest.
